# Giant epidermal cyst in the posterior neck developing over 40 years: A case report

**DOI:** 10.3892/etm.2013.1383

**Published:** 2013-11-06

**Authors:** TAE WON PARK, JONG KIL KIM, JUNG RYUL KIM

**Affiliations:** 1 Department of Psychiatry, Chonbuk National University Medical School, Jeonju, Jeonbuk 561-756, Republic of Korea; 2 Research Institute of Clinical Medicine, Chonbuk National University Medical School, Jeonju, Jeonbuk 561-756, Republic of Korea; 3 Department of Orthopaedic Surgery, Chonbuk National University Medical School, Jeonju, Jeonbuk 561-756, Republic of Korea

**Keywords:** giant epidermal cyst, posterior neck, psychiatric symptom, cosmetic problem

## Abstract

Conventional epidermal cysts are generally small, slow-growing, non-tender, dome-shaped lesions. An epidermal cyst is usually asymptomatic until it is infected or enlarged to the extent that it causes damage to adjacent anatomical structures. However, few cases of giant epidermal cysts in the neck have been reported. The present case reports a giant epidermal cyst in the posterior neck, which grew to an extremely large size for >40 years without inflammation or rupture, and was misdiagnosed as a large soft tissue neoplasm. The patient exhibited depression and developed social anxiety due to the negative cosmetic consequences of the large mass. The patient underwent excision of the mass. At the follow-up examination two years postoperatively, there were no local recurrence and the psychiatric symptoms of the patient were completely resolved. To the best of our knowledge, a giant epidermal cyst growing for >40 years has not previously been reported.

## Introduction

Epidermal cysts are the most common benign lesions. They usually appear in hair-bearing skin areas, including the scalp, face, neck, back and trunk (1). Conventional epidermal cysts are generally small, slow-growing, non-tender, dome-shaped lesions. An epidermal cyst is usually asymptomatic unless it becomes infected or is enlarged to the extent that it causes damage to adjacent anatomical structures. A number of authors have previously reported giant epidermal cysts with diameters >5 cm (2,3). However, few cases of giant epidermal cysts in the neck have been reported. The present study concerns a giant epidermal cyst present in the posterior neck, which grew to an extremely large size for >40 years without inflammation or rupture, and was misdiagnosed as a large soft tissue neoplasm. The patient exhibited depression and developed social anxiety due to the negative cosmetic consequences of the huge mass. To the best of our knowledge, a giant epidermal cyst growing for >40 years has not previously been reported. The study was approved by the Ethics Committee of Chonbuk National University Hospital. Informed consent was obtained from the patient and the family of the patient.

## Case Report

**Figure 1:**
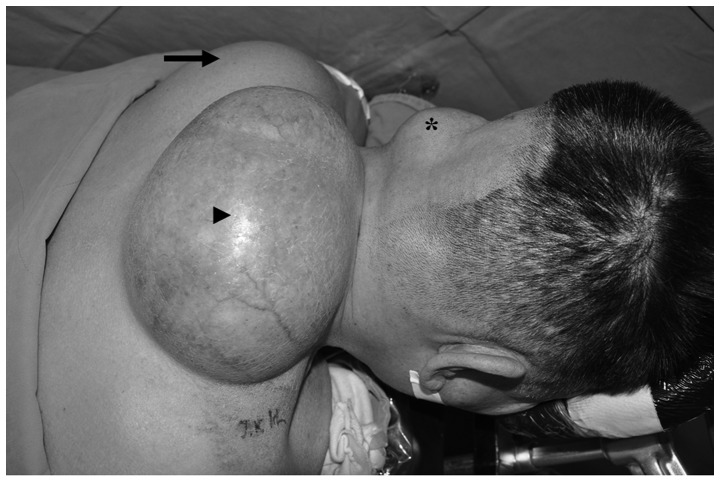
Three giant subcutaneous masses in the posterior neck (arrowhead), left upper back (arrow), and left scalp (asterisk).

A 66-year-old male presented to The Department of Orthopaedic Surgery, Chonbuk National University Medical School (Jeonju, Korea) with three soft painless masses in the posterior neck, left upper back and left scalp. The patient initially became aware of the large mass in the midline of the posterior neck ~40 years previously and the other masses ~5 years previously. The masses had gradually become enlarged. Clinical examination revealed that all masses were soft, movable and well defined (Fig. 1). There was no history of trauma or any previous surgery. The patient presented with discomfort due to compression from the extremely large tumor. Moreover, the patient exhibited depression and had developed social anxiety as a result of the cosmetic problems caused by the large mass in the posterior neck. Consequently, the patient was prescribed several antidepressants and anti-anxiety drugs by an outpatient psychiatric clinic.

**Figure 2:**
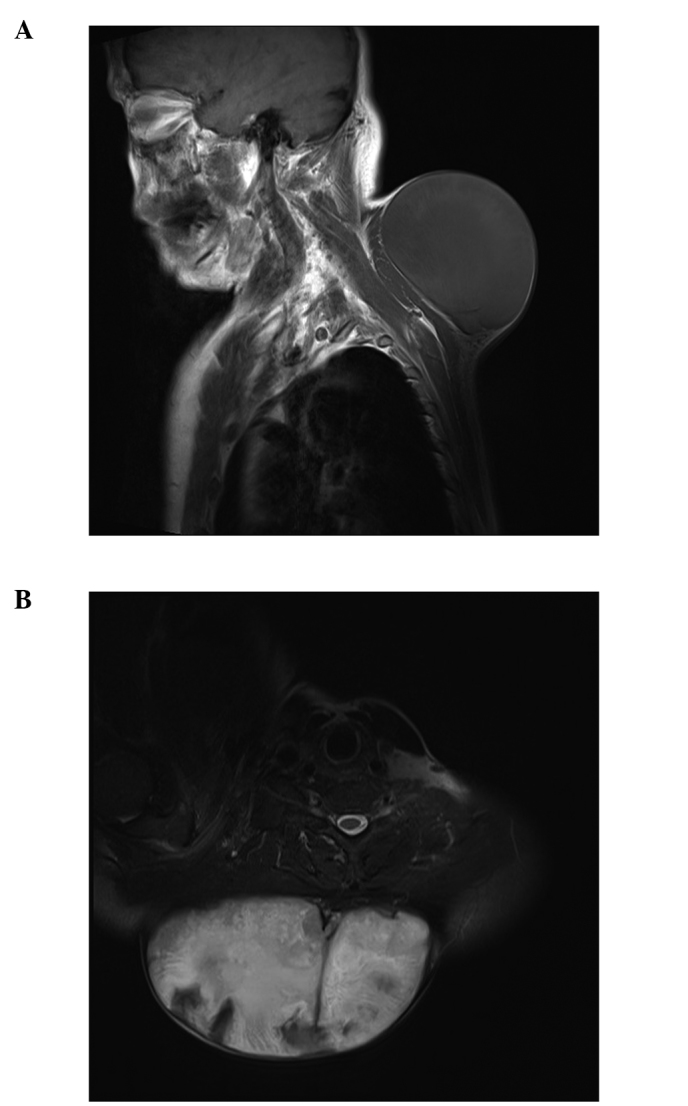
(A) Sagittal T1-weighted MRI revealed a homogenous low intermediate signal intensity mass, surrounded by a low signal intensity well-marginated capsule in the subcutaneous fat of the posterior neck. (B) Axial T2-weighted MRI images showed an oval mass with high signal intensity and debris with low signal intensity at the surrounding capsule.

Magnetic resonance imaging (MRI) revealed a large encapsulated homogeneous mass measuring 18×21×15 cm in the midline of the posterior neck. The mass had low signal intensity on T1-weighted images, while in T2-weighted images, its signals were of high signal intensity with multiple occurrences of focal low signal intensity debris (Fig. 2). The masses in the left upper back (13×12×6 cm) and the left scalp (5×5×4 cm) were diagnosed as subcutaneous lipomas based on MRI findings.

**Figure 3:**
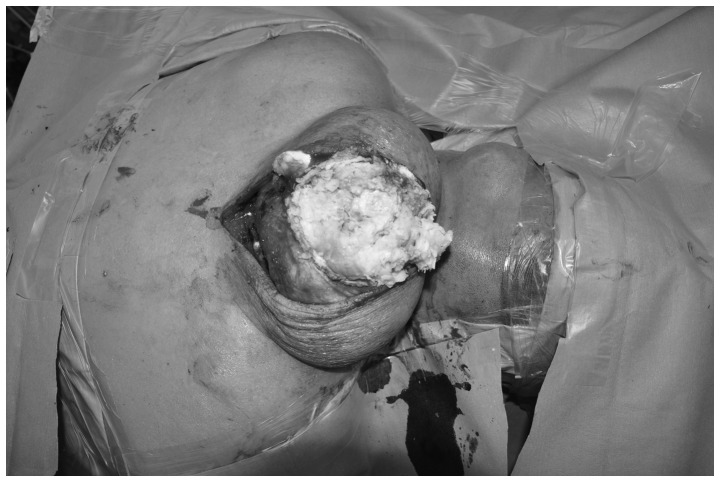
Mass in the posterior neck was well defined with an elastic texture and contained a cream-colored fluid with a butter-like consistency.

The patient underwent excision of all masses under general anesthesia. The mass in the posterior neck was well defined with an elastic texture and contained a cream-colored fluid with the consistency of butter (Fig. 3). The other masses contained well-capsulated mature fat tissue. A skin graft of superficial thickness was performed to correct skin defects following the excision of friable skin from the epidermal cyst of the posterior neck.

**Figure 4:**
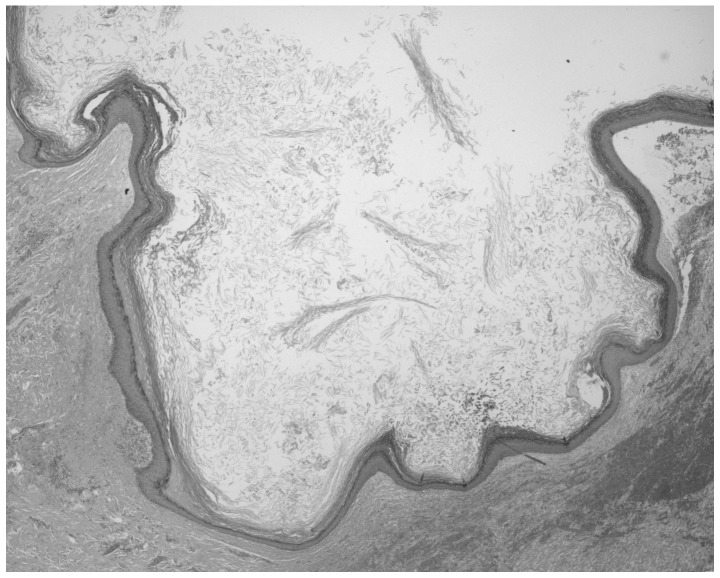
Histopathological examination showed the epidermal cyst wall with a thin layer of benign stratified squamous epithelium and lamellated keratin debris present in the cyst (H&E-stained; original magnification, ×20).

Histopathological examination revealed an epidermal cyst wall with a thin layer of benign stratified squamous epithelium and lamellated keratin debris present within the cyst (Fig. 4).

**Figure 5:**
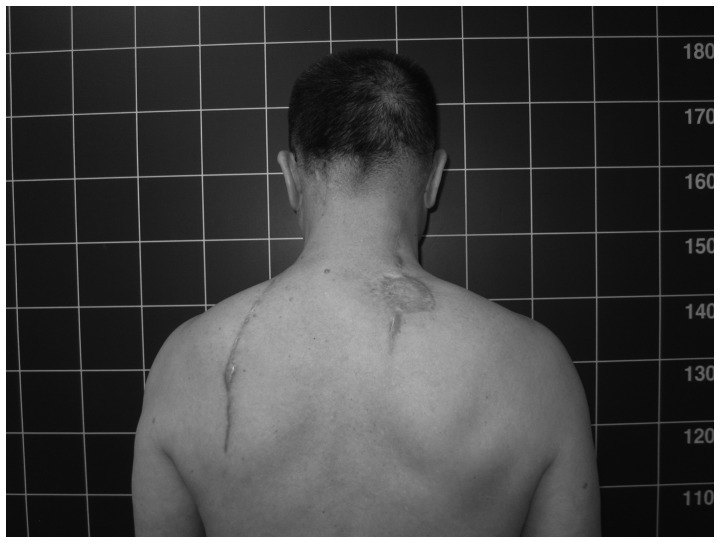
A photograph at the two-year postoperative follow-up showed excellent cosmetic results without recurrence.

At the follow-up examination two years postoperatively, there were no local recurrences of the lesions and the psychiatric symptoms of the patient were completely resolved (Fig. 5).

## Discussion

Epidermoid cysts are characteristically observed in the trunk, neck and face, and are likely to be derived from the inflammation of pilosebaceous structures (2). Cysts on the acral surfaces of the skin are considered to arise from the implantation of epidermis into the dermis through trauma. These cysts usually grow through the accumulation of epithelial and keratinous debris (3,4). However, an epidermal cyst with a diameter ≥5 cm is rare. Giant epidermal cysts in the head and neck present several problems. Firstly, they are easy to rupture and this may induce infection. Secondly, giant epidermal cysts may compress adjacent organs, including major arteries, veins and nerves (2,4). Furthermore, giant masses may cause cosmetic problems due to the high visibility of the head and neck. The patient in the present case exhibited pressure symptoms as well as psychiatric symptoms, including depression and social anxiety, caused by the cosmetic appearance of the large mass.

A proliferative epidermal cyst >5 cm in size is locally aggressive and may potentially be a malignant tumor. The development of squamous cell carcinoma in epidermal cysts is rare and few cases have been reported (5). In a proliferative epidermal cyst, epithelial proliferation from the cyst wall projects into the lumen (5). Generally, squamous cell carcinomas in epidermal cysts have a low malignant potential (5). No malignant transformations were identified in this case.

The present case of a giant epidermal cyst was associated with two large adjacent lipomas. There was no clear association between the epidermal cyst and the lipomas. To the best of our knowledge, this case is the first report of a giant epidermal cyst associated with two separate large lipomas in the head and neck.

In conclusion, the present case demonstrates that giant epidermal cysts may grow for long durations of time and produce adverse effects due to the pressure exerted on surrounding structures, as well as serious cosmetic problems that may require psychiatric medication. Therefore, early surgical excision is recommended for patients exhibiting giant epidermal cysts.
